# Supplementary material for: Antibody–Drug Conjugate Made of Zoledronic Acid and the Anti-CD30 Brentuximab–Vedotin Exert Anti-Lymphoma and Immunostimulating Effects
Source: Cells. 2024 May 17;13(10):862. doi: 10.3390/cells13100862 (PMC11119185; doi:10.3390/cells13100862)
Supplement: Supplementary file 1 [file cells-13-00862-s001.zip › cells-3007595-supplementary.pdf]

Supplementary material

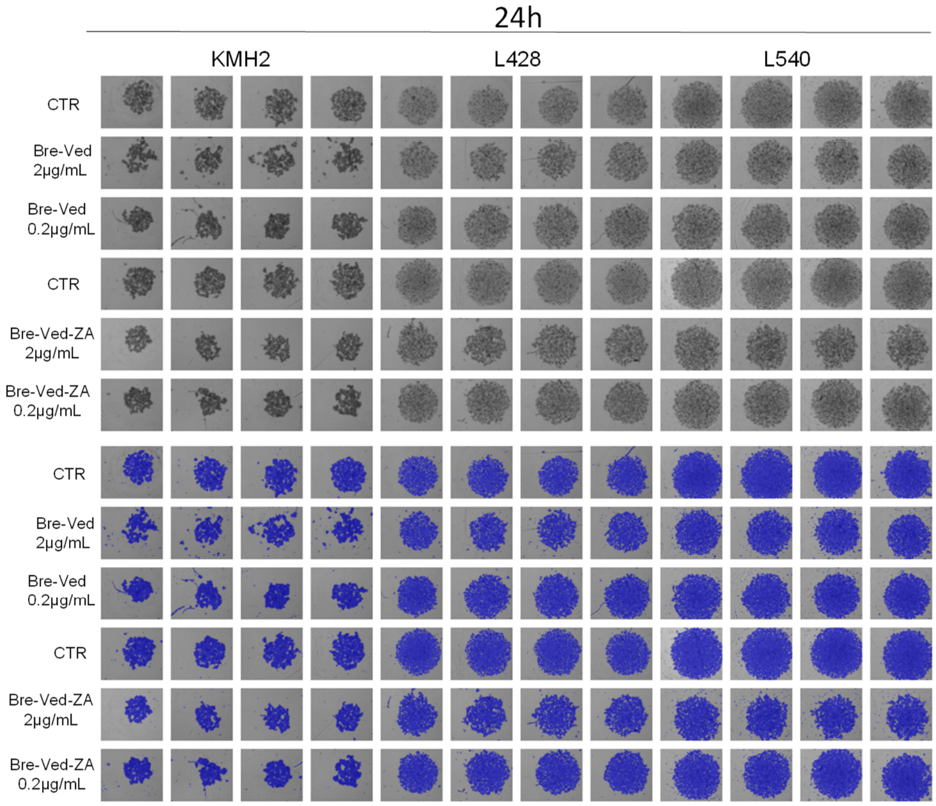

Supplemental Figure S1. Analysis and masking with CellStudio software of the cell area of HL cell lines upon culture. $5x10^3$ cells were cultured in quadruplicate U-bottomed wells (KMH2, L428 and L540) for 24h and image of each well was taken automatically by CELLCYTE X™ cell culture scanner (upper grey images). Afterwards, the images were analysed using the CELLSTUDIO software to determine the area occupied by the growing cells (lower blue images). The cell area size in mm^2 was calculated automatically and plotted as shown in figure 6B and 6C. Images of the same wells were taken at different time points as illustrated in the suppl. Figures S2, 3 and 4.

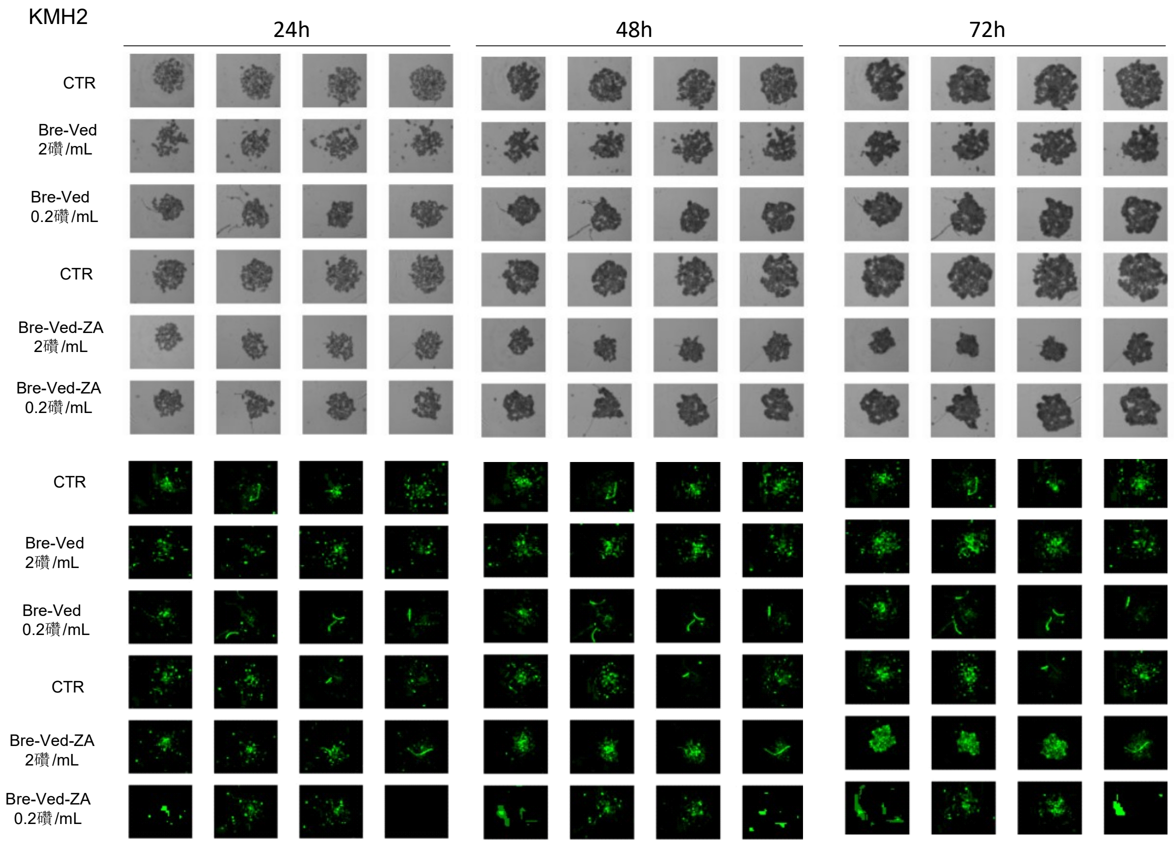

Supplemental Figure S2. Cultures of the KMH2 HL cell line with anti-CD30 ADC. $5x10^3$ KMH2 cells were cultured in quadruplicate U-bottomed wells for 24, 48 and 72h at 37°C, 5% CO_2 and images of each well were taken automatically by CELLCYTE X™ cell culture scanner (upper grey images). Afterwards, the images were analysed using the CELLSTUDIO software to determine the area covered by the growing cells. The cell area size in mm^2 was calculated automatically and plotted as shown in figure 6B. The same wells were incubated with the C.LIVE Tox green probe labelling dying and died cells at the same time points. The level of green fluorescence in each well was calculated automatically by the CELLSTUDIO software in arbitrary units and plotted as shown in figure 7 panel B.

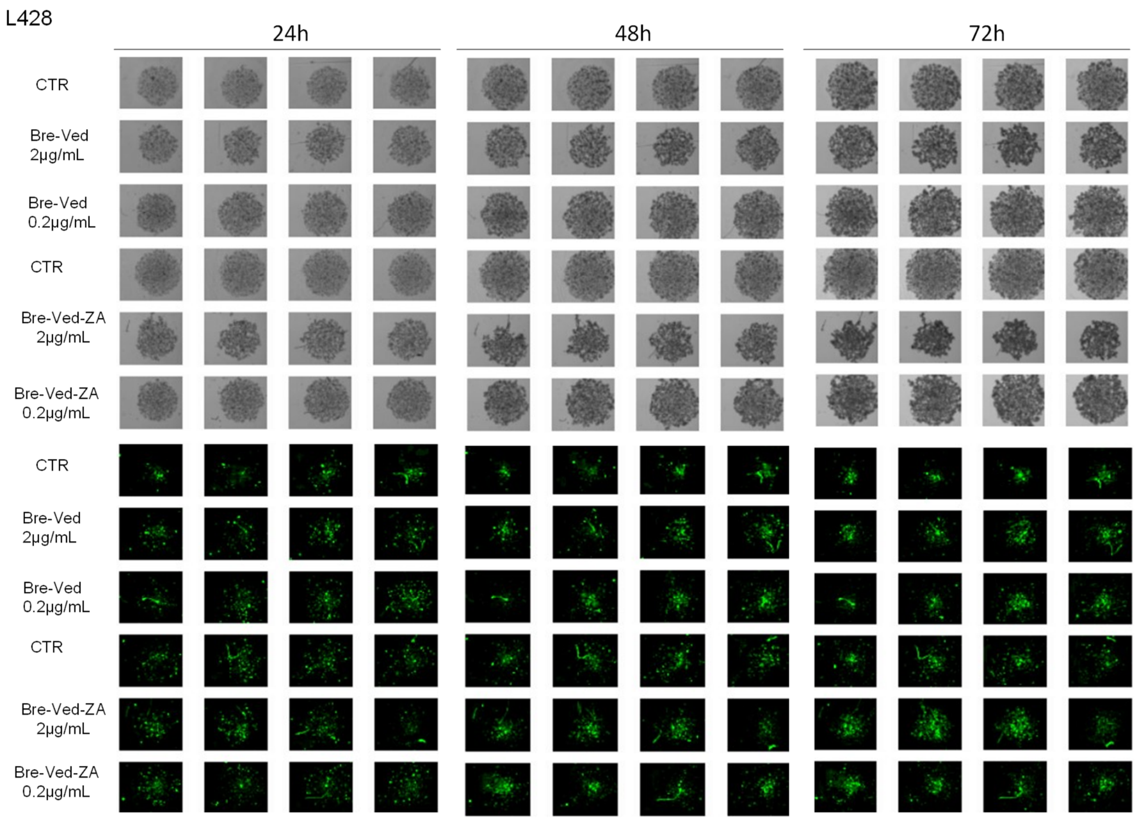

Supplemental Figure S3. Cultures of the L428 HL cell line with anti-CD30 ADC. 5x10^3 L428 cells were cultured in quadruplicate U-bottomed wells for 24, 48 and 72h at 37°C, 5% CO_2 and images of each well were taken automatically by CELLCYTE X™ cell culture scanner (upper grey images). Afterwards, the images were analysed using the CellStudio software to determine the area covered by the growing cells. The cell area size in mm^2 was calculated automatically and plotted as shown in figure 6C upper plot. The same wells were incubated with the C.LIVE Tox green probe labelling dying and died cells at the same time points. The level of green fluorescence in each well was calculated automatically by the CELLSTUDIO software in arbitrary units and plotted as shown in figure 7 panel C upper plot.

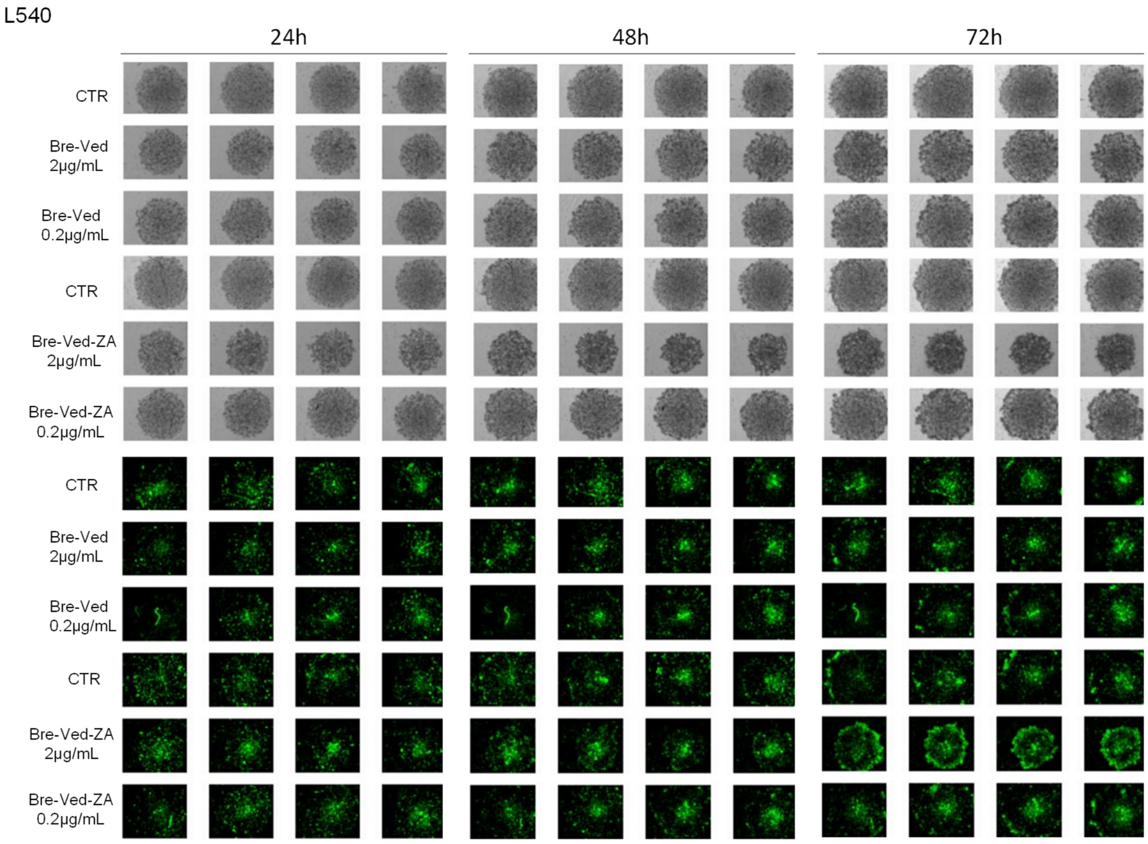

Supplemental Figure S4. Cultures of the L540 HL cell line with anti-CD30 ADC. $5x10^3$ L540 cells were cultured in quadruplicate U-bottomed wells for 24, 48 and 72h at 37°C, 5% CO_2 and images of each well were taken automatically by CELLCYTE X™ cell culture scanner (upper grey images). Afterwards, the images were analysed using the CELLSTUDIO software to determine the area covered by the growing cells. The cell area size in mm^2 was calculated automatically and plotted as shown in figure 6C lower plot. The same wells were incubated with the C.LIVE Tox green probe labelling dying and died cells at the same time points. The level of green fluorescence in each well was calculated automatically by the CELLSTUDIO software in arbitrary units and plotted as shown in figure 7 panel C lower plot.

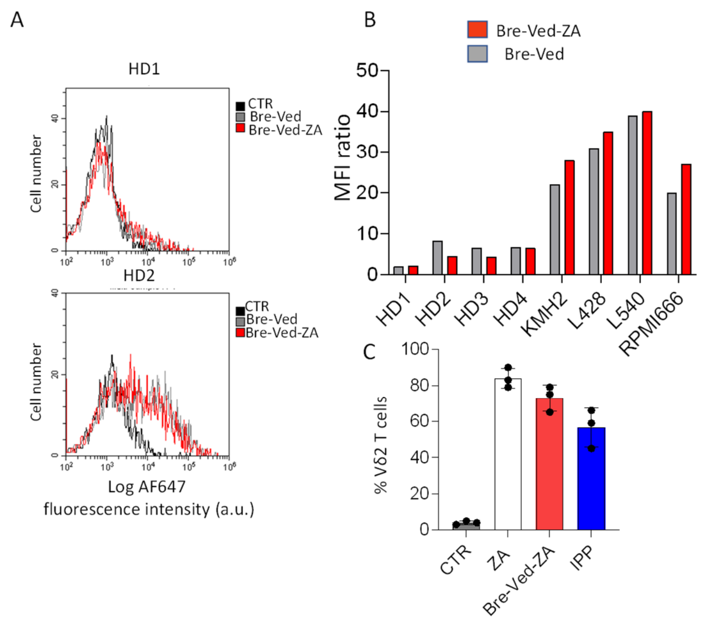

Supplemental Figure S5. Vδ2 T cell reactivity with the anti-CD30 therapeutic ADC. A. Vδ2 T cells were stained with anti-CD30 Bre-Ved (grey) or Bre-Ved-ZA (red) antibody followed by the anti-human AlexaFluor647 antiserum. CTR: cells stained with the second reagent alone (black). The results are expressed as cell number vs Log AF647 fluorescence intensity in arbitrary unit (a.u.). B. Mean fluorescence ratios between the MFI of Bre-Ved (grey) or Bre-Ved-ZA (red) and cells stained with the second reagent alone. Results are from 4 Vδ2 T cell populations at day 14 from healthy donors (HD) and from the indicated CD30+ HL (KMH2, L428, L540) and lymphoblastoid tumor cells (RPMI6666). C. Expansion of Vδ2 T cells evaluated by immunofluorescence at day 14 upon stimulation of PBMC at the onset of the assay with either 1μM soluble ZA (white), or 2μg/mL of Bre-Ved-ZA (red) or 1μM soluble IPP (blue). CTR: Vδ2 T cells present at the onset of the assay. Results are shown as percentage of Vδ2 T cells and are the mean±SD of three experiments with three HD.
